# Bioreducible Polymer Micelles Based on Acid-Degradable Poly(ethylene glycol)-poly(amino ketal) Enhance the Stromal Cell-Derived Factor-1α Gene Transfection Efficacy and Therapeutic Angiogenesis of Human Adipose-Derived Stem Cells

**DOI:** 10.3390/ijms19020529

**Published:** 2018-02-09

**Authors:** Tae-Jin Lee, Min Suk Shim, Taekyung Yu, Kyunghee Choi, Dong-Ik Kim, Soo-Hong Lee, Suk Ho Bhang

**Affiliations:** 1 School of Chemical Engineering, Sungkyunkwan University, Suwon 16419, Korea; eclatwiz@hotmail.com; 2 Division of Bioengineering, Incheon National University, Incheon 406-772, Korea; msshim@inu.ac.kr; 3 Department of Chemical Engineering, College of Engineering, Kyung Hee University, Youngin 17104, Korea; tkyu@khu.ac.kr; 4 Department of Pathology and Immunology, Washington University School of Medicine, St. Louis, MO 63110, USA; kchoi@wustl.edu; 5 Developmental, Regenerative, and Stem Cell Biology Program, Washington University School of Medicine, St. Louis, MO 63110, USA; 6 Division of Vascular Surgery, Samsung Medical Center, Sungkyunkwan University School of Medicine, Seoul 06351, Korea; dikim@skku.edu; 7 Department of Biomedical Science, CHA University, Seongnam 463-400, Korea

**Keywords:** angiogenesis, bioreducible polymer, gene therapy, hADSCs, *SDF-1α*

## Abstract

Adipose-derived stem cells (ADSCs) have the potential to treat ischemic diseases. In general, ADSCs facilitate angiogenesis by secreting various pro-angiogenic growth factors. However, transplanted ADSCs have a low therapeutic efficacy in ischemic tissues due to their poor engraftment and low viability. Stromal cell-derived factor-1α (SDF-1α) improves the survival rate of stem cells transplanted into ischemic regions. In this study, we developed acid-degradable poly(ethylene glycol)-poly(amino ketal) (PEG-PAK)-based micelles for efficient intracellular delivery of *SDF-1α* plasmid DNA. The *SDF-1α gene* was successfully delivered into human ADSCs (hADSCs) using PEG-PAK micelles. Transfection of *SDF-1α* increased *SDF-1α*, vascular endothelial growth factor, and basic fibroblast growth factor gene expression and decreased apoptotic activity in hADSCs cultured under hypoxic conditions in comparison with conventional gene transfection using polyethylenimine. *SDF-1α*-transfected hADSCs also showed significantly increased *SDF-1α* and VEGF expression together with reduced apoptotic activity at 4 weeks after transplantation into mouse ischemic hindlimbs. Consequently, these cells improved angiogenesis in ischemic hindlimb regions. These PEG-PAK micelles may lead to the development of a novel therapeutic modality for ischemic diseases based on an acid-degradable polymer specialized for gene delivery.

## 1. Introduction

Adipose-derived stem cells (ADSCs) have the potential to treat ischemic diseases. These cells facilitate angiogenesis by secreting various pro-angiogenic growth factors such as vascular endothelial growth factor (VEGF) and fibroblast growth factor (FGF) [1]. Moreover, ADSCs can be easily obtained from adipose tissues [1]. However, ADSCs transplanted into ischemic region have a low therapeutic efficacy due to their poor engraftment and low viability [2]. To improve the therapeutic efficacy, the survival of ADSCs must be enhanced, which would increase the secretion of paracrine pro-angiogenic factors. 

Ischemic tissues secrete various cytokines, chemokines, proteins, and growth factors [3]. Among these, stromal cell-derived factor-1α (SDF-1α) induces the recruitment and migration of stem cells [3]. SDF-1α can enhance cell survival by inactivating the cell death molecular pathway and increasing the transcription of cell survival genes [4,5]. Moreover, SDF-1α improves the survival rate of transplanted mesenchymal stem cells (MSCs) [6,7]. Cationic polymer-based nanoparticles have been researched for gene delivery [8]. Polyethylenimine (PEI), a polycation with a high cationic charge density, is a highly efficient gene delivery vector [8]. Cationic polymers can condense negatively charged nucleic acids and protect them from nuclease-induced degradation [9]. However, high-molecular-weight PEI is associated with problems such as non-biodegradability and high cytotoxicity [10]. To overcome these problems and increase the efficiency of gene delivery, biodegradable and nontoxic polymers that can efficiently release nucleic acids in response to cellular stimuli are required [11].

In this study, poly(ethylene glycol)-poly(amino ketal) (PEG-PAK), an acid-degradable cationic polymer whose backbone contains ketal linkages, was used to deliver *SDF-1α* plasmid DNA (pDNA) into human adipose-derived stem cells (hADSCs). PEG-PAK micelles efficiently delivered *SDF-1α* pDNA into the cytoplasm of hADSCs in comparison with conventional gene carriers and exhibited significantly reduced cytotoxicity. When transplanted into the ischemic hindlimbs of mice, *SDF-1α*-transfected hADSCs demonstrated improved viability and therapeutic angiogenesis. These PEG-PAK micelles may lead to the development of a novel therapeutic modality for ischemic diseases based on an acid-degradable polymer specialized for gene delivery.

## 2. Results

### 2.1. Characterization and Gene Transfection Efficiency of PEG-PAK Micelles

**Figure 1:**
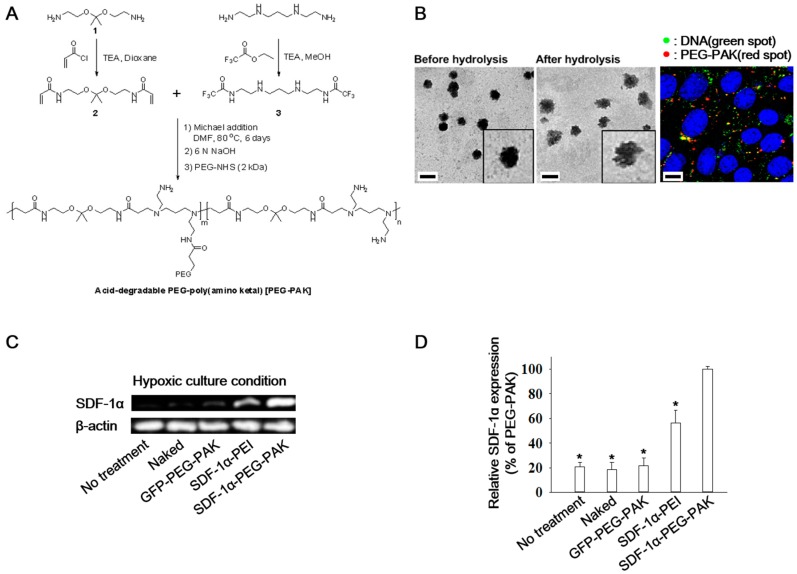
(**A**) Scheme of the synthesis of acid-degradable PEG-PAK; (**B**) Transmission electron microscopy images of *SDF-1α*/PEG-PAK micelles before and after acid hydrolysis (pH 5.0 for 8 h at 37 °C, scale bars indicate 200 nm) and confocal laser scanning microscopy images showing the intracellular colocalization of Alexa Fluor 488-labeled *SDF-1α* pDNA (green) and Cy3-labeled PEG-PAK (red) in hADSCs (blue indicates nuclei stained with DRAQ5, scale bar indicates 10 μm); (**C**) RT-PCR analysis and (**D**) quantification of SDF-1α expression in hADSCs transfected with SDF-1α using various methods under hypoxic culture conditions (* *p* < 0.05 compared with SDF-1α-PEG-PAK group). PEG-PAK: poly(ethylene glycol)-poly(amino ketal); SDF-1α: stromal cell-derived factor-1α; RT-PCR: reverse transcription-polymerase chain reaction; hADSCs: human adipose-derived stem cells; GFP: green fluorescence protein.

Acid-degradable poly(amino ketal) (PAK) was synthesized for efficient DNA delivery (Figure 1A). Before acid hydrolysis, pDNA/PEG-PAK micelles were spherical (Figure 1B, left). After 8h of acid hydrolysis, the structure of pDNA/PEG-PAK micelles was loosened and disrupted (Figure 1B, right). Intracellular dissociation of pDNA from pDNA/PEG-PAK micelles was confirmed by confocal laser scanning microscopy. A high level of pDNA (green dots) dissociated from PEG-PAK micelles (red dots) was found in the cytosol and perinuclear region (Figure 1B). The gene transfection efficiency of *SDF-1α* pDNA/PEG-PAK micelles was evaluated by incubating them with hADSCs for 48 h under hypoxic conditions. *SDF-1α* mRNA expression was measured and quantified (Figure 1C,D). mRNA expression of *SDF-1α* was significantly higher in the SDF-1α-PEG-PAK group than in the other groups.

### 2.2. Decreased Apoptosis and Enhanced Secretion of Pro-Angiogenic Factors in hADSCs Overexpressing SDF-1α 

**Figure 2:**
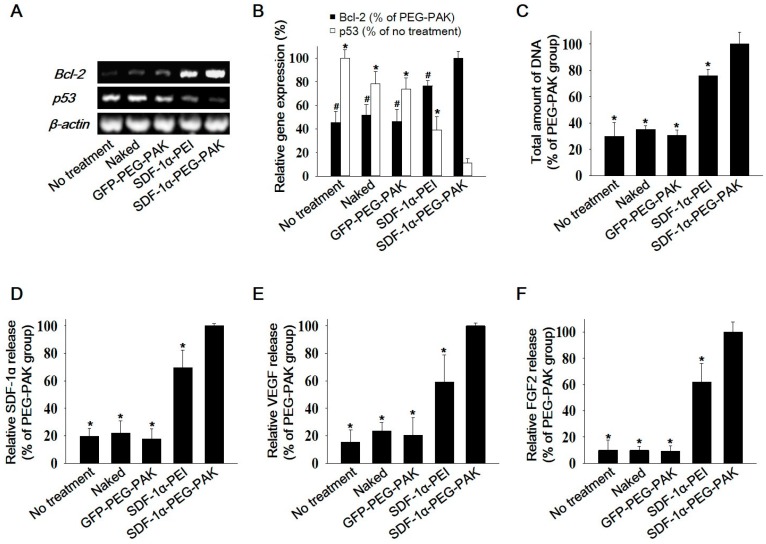
Apoptotic activity and pro-angiogenic growth factor secretion in hADSCs transfected with SDF-1α using PEG-PAK micelles. (**A**) RT-PCR analysis of the anti-apoptotic factor *Bcl-2* and the pro-apoptotic factor *p53* and (**B**) quantification of their expression in hADSCs transfected with *SDF-1α* using various methods. (**C**) Total amount of DNA in each group showing relative cell viability. Relative levels of (**D**) SDF-1α, (**E**) VEGF, and (**F**) FGF2 secretion by hADSCs transfected with *SDF-1α* using various methods. Secretion was quantified via enzyme-linked immunosorbent assays. (*^,#^ *p* < 0.05 compared with SDF-1α-PEG-PAK group). VEGF: vascular endothelial growth factor; FGF2: basic fibroblast growth factor.

The anti-apoptotic effect of *SDF-1α* overexpression using PEG-PAK micelles was investigated in hADSCs cultured under hypoxic conditions. Expression of the anti-apoptotic gene *Bcl-2* and the pro-apoptotic gene *p53* was quantified by reverse transcription-polymerase chain reaction (RT-PCR) (Figure 2A,B). *Bcl-2* and *p53* expression was increased and decreased, respectively, in hADSCs transfected with *SDF-1α* pDNA/PEG-PAK micelles. The total amount of DNA was higher in these cells than in the other groups (Figure 2C). Moreover, hADSCs transfected with SDF-1α pDNA/PEG-PAK micelles secreted higher levels of *SDF-1α*, VEGF, and FGF2 than cells in the other groups (Figure 2D–F).

### 2.3. Effect of hADSCs Transfected with SDF-1α pDNA/PEG-PAK Micelles in Ischemic Limbs

**Figure 3:**
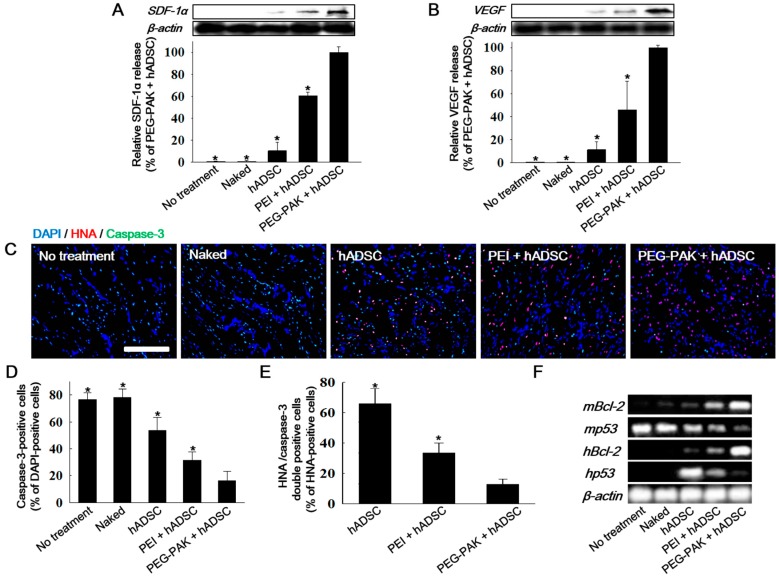
Western blot analysis and quantification of (**A**) SDF-1α and (**B**) VEGF expression in the mouse hindlimb ischemia model 3 days after the various treatments; (**C**) Immunofluorescence staining of caspase-3 (green) and HNA (red) in ischemic limb tissues retrieved 3 days after treatment (blue indicates nuclei stained with 4′,6-diamidino-2-phenylindole (DAPI), scale bar = 100 μm). Percentages of (**D**) caspase-3-positive cells (apoptotic cells) among DAPI-positive cells (total cells) and (**E**) HNA/caspase-3 double-positive cells (apoptotic hADSCs) among HNA-positive cells (hADSCs) in the ischemic region (* *p* < 0.05 compared with PEG-PAK + hADSC group); (**F**) RT-PCR analysis of human and mouse *Bcl-2* (anti-apoptotic factor) and *p53* (pro-apoptotic factor) in ischemic limbs.

The therapeutic efficacy of hADSCs transfected with *SDF-1α* pDNA/PEG-PAK micelles was evaluated in a mouse hindlimb ischemia model. After induction of ischemia, the mice were treated with hADSCs or those transfected with *SDF-1α* pDNA/PEG-PAK micelles (PEG-PAK + hADSC), *SDF-1α* pDNA/PEI polyplexes (PEI + hADSC), or naked *SDF-1α* pDNA (naked). Mice with ischemic injury were also injected with phosphate-buffered saline (PBS) as a control (no treatment). *SDF-1α* expression in ischemic limbs was significantly increased in the PEG-PAK + hADSC group at 21 days after treatment (Figure 3A). Consistently, VEGF expression was also increased in this group (Figure 3B). 

Cell survival in ischemic limbs was investigated by double immunofluorescence staining of caspase-3 and human nuclear antigen (HNA) (Figure 3C). There were fewer caspase-3-positive cells (apoptotic cells in the ischemic limb) and HNA/caspase-3 double-positive cells (apoptotic hADSCs) in the PEG-PAK + hADSC group than in the other groups (Figure 3C–E). Moreover, mRNA expression of human *Bcl-2* and *p53* was higher and lower, respectively, in the PEG-PAK + hADSC group than in the PEI + hADSC, hADSC, and naked groups (Figure 3F). Similarly, mRNA expression of mouse *Bcl-2* and *p53* expression was higher and lower, respectively, in the PEG-PAK + hADSC group than in the other groups (Figure 3F).

### 2.4. In Vivo Pro-Angiogenic Effect of hADSCs Transfected with SDF-1α pDNA/PEG-PAK Micelles

**Figure 4:**
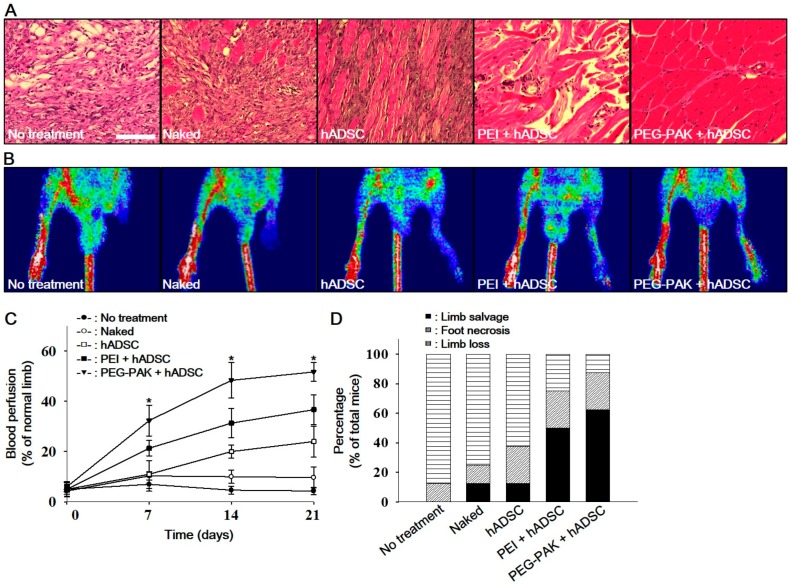
(**A**) Hematoxylin and eosin staining of hindlimb tissues obtained 21 days after ischemic injury (scale bars indicate 100 µm). (**B**) Representative laser Doppler perfusion imaging analysis performed at 21 days after treatment. (**C**) Blood perfusion of ischemic limbs relative to that of normal limbs at 0, 7, 14, and 21 days after treatment (* *p* < 0.01 compared with other groups). (**D**) Percentage of mice displaying limb salvage at 21 days after treatment.

**Figure 5:**
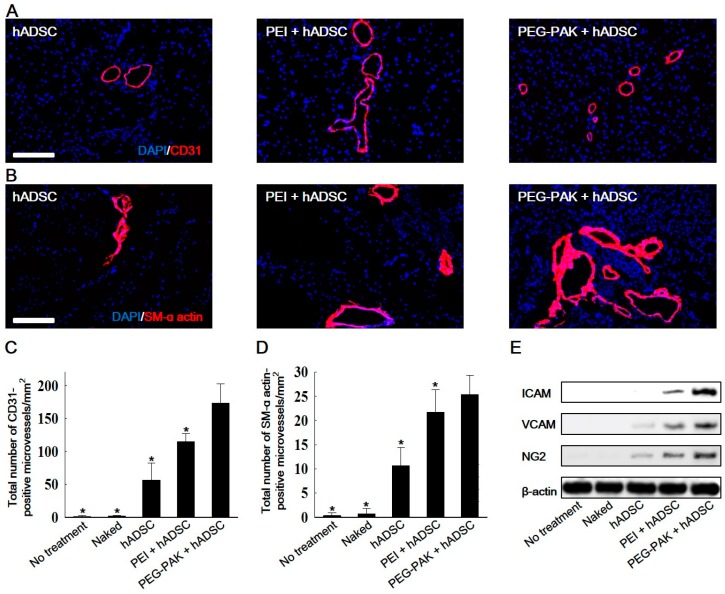

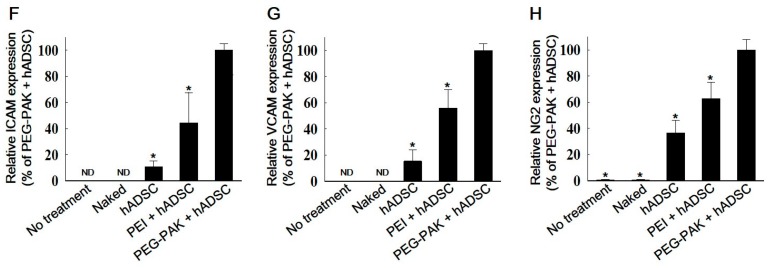
Representative immunohistochemical images of microvessels positive for (**A**) CD31 (red) and (**B**) smooth muscle (SM) α-actin (red) retrieved from hindlimb tissues 21 days after treatment (blue indicates nuclei stained with DAPI, scale bars indicate 100 µm). Quantification of microvessels positive for (**C**) CD31 and (**D**) SM α-actin in hindlimb tissues (* *p* < 0.05 compared with PEG-PAK + hADSC group); (**E**) Western blot analysis and quantification of (**F**) ICAM, (**G**) VCAM, and (**H**) NG2 expression in hindlimb tissues at 21 days after treatment. ICAM: intercellular adhesion molecule; VCAM: vascular cell adhesion molecule.

**Figure 6:**
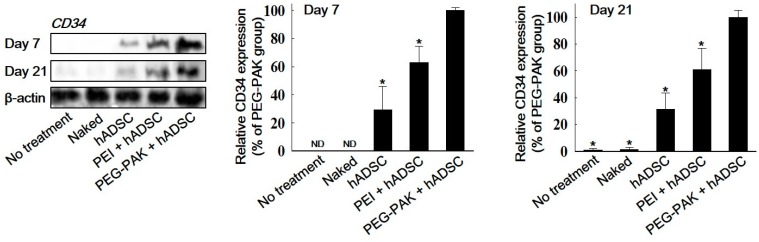
Western blot analysis and quantification of CD34 expression in ischemic hindlimb tissues at 7 and 21 days after treatment (* *p* < 0.05 compared with PEG-PAK + hADSC group).

Fibrotic tissue formation in ischemic hindlimb regions was reduced in the PEG-PAK + hADSC group (Figure 4). Moreover, blood perfusion in ischemic limbs was significantly higher in the PEG-PAK + hADSC group than in the other groups (Figure 4B,C). Furthermore, limb salvage was observed in 60% of mice in the PEG-PAK + hADSC group (Figure 4D). The density of CD31-positive microvessels was significantly higher in the PEG-PAK + hADSC group than in the other groups at 21 days after treatment (Figure 5A,C). Moreover, the density of smooth muscle (SM)-α actin-positive vessels was significantly higher in the PEG-PAK + hADSC group than in the other groups (Figure 5B,D). Transplantation of hADSCs transfected with *SDF-1α* pDNA/PEG-PAK micelles increased expression of the proteoglycan NG2, a marker of pericytes, in ischemic limbs, which was related to the stabilization of microvessels (Figure 5E,H). Expression of intercellular adhesion molecule (ICAM) and vascular cell adhesion molecule (VCAM) was also increased in the PEG-PAK + hADSC group (Figure 5E,H). This indicates that the number of activated endothelial cells was increased, which may promote the recruitment of endothelial progenitor cells (EPCs) into ischemic regions. Indeed, CD34 expression in ischemic limbs was higher in the PEG-PAK + hADSC group than in the other groups, providing strong evidence that the recruitment of EPCs into ischemic regions was increased (Figure 6).

## 3. Discussion 

One of the major obstacles in stem cell-based therapy is the poor engraftment and low viability of transplanted stem cells, leading to a low therapeutic efficacy [2,12,13]. Various attempts have been made to overcome this problem [14,15,16,17]. Gene modifications, including overexpression of the serine/threonine protein kinase Akt, the proto-oncogene with serine/threonine-protein kinase activity Pim-1, or heme oxygenase-1, have been used to improve the survival of transplanted cells [18,19,20]. Along with these anti-apoptotic genes, expression of the well-known pro-angiogenic factor SDF-1α also improves the survival of transplanted stem cells such as MSCs and EPCs [6,21]. Therefore, we hypothesized that transfection of *SDF-1α* would enhance the survival and therapeutic efficacy of hADSCs. As expected, *SDF-1α* transfection decreased apoptotic activity in hADSCs cultured under hypoxic conditions (Figure 2) and transplanted into ischemic regions (Figure 3). Moreover, VEGF secretion was enhanced in *SDF-1α*-transfected hADSCs (Figure 2 and Figure 3). This result shows that SDF-1α released from transplanted hADSCs transfected with SDF-1α pDNA/PEG-PAK micelles stimulated VEGF secretion [6,22]. Along with the improved survival of transplanted hADSCs, their enhanced secretion of pro-angiogenic factors might help to decrease ischemic damage and improve angiogenesis (Figure 4). 

Recruitment of EPCs by SDF-1α-transfected hADSCs enhanced the angiogenic efficacy. EPC recruitment into ischemic regions, as represented by CD34 expression in ischemic limbs, was enhanced at 7 and 21 days following transplantation of SDF-1α-transfected hADSCs (Figure 6). SDF-1α release in ischemic limbs was increased by transplantation of hADSCs and was further amplified by transplantation of *SDF-1α*-transfected hADSCs (Figure 3). In addition, transplantation of SDF-1α-transfected hADSCs increased expression of ICAM and VCAM in ischemic tissues (Figure 5E–H). SDF-1a is a major chemoattractant for EPCs [23], and local release of SDF-1α increases homing of these cells [24]. ICAM and VCAM also contribute to EPC homing [25,26]. These results suggest that the increases in SDF-1α, ICAM, and VCAM expression upon transplantation of *SDF-1α*-transfected hADSCs led to the recruitment of EPCs, which facilitated angiogenesis in ischemic regions.

In summary, we have developed an acid-degradable non-viral carrier for efficient intracellular delivery. PEG-PAK micelles successfully delivered SDF-1α pDNA into hADSCs. SDF-1α-transfected hADSCs demonstrated increased SDF-1α, VEGF, and FGF2 expression and decreased apoptotic activity when cultured under hypoxic conditions. Similarly, SDF-1α-transfected hADSCs exhibited enhanced SDF-1α and VEGF expression and decreased apoptotic activity when transplanted into ischemic tissues. Finally, the enhanced viability of hADSCs increased the angiogenic efficacy in ischemic tissues. This study may offer a clever strategy to treat ischemic diseases using an acid-degradable polymer specialized for gene delivery.

## 4. Materials and Methods

### 4.1. Materials

Acryloyl chloride (C_3_H_3_ClO, Ward Hill, MA, USA) was obtained from Alfa Aesar. *N*,*N*′-*bis*(2-aminoethyl)-1,3-propanediamine, ethyl trifluoroacetate(C_4_H_5_F_3_O_2_), 25 kDa branched polyethylenimine (B-PEI), trimethylamine(C_3_H_9_N), 3-(4,5-dimethyl-2-thiazolyl)-2,5-diphenyltetrazoliumbromide (MTT), and sodium hydroxide were prepared from Sigma Aldrich (St. Louis, MO, USA). PD-10 desalting columns were purchased from GE Healthcare (Piscataway, NJ, USA). Alexa Fluor 488 nucleic acid-labeling kit were purchased from Invitrogen (Eugene, OR, USA). 

### 4.2. Synthesis of Acid-Degradable (PEG-PAK) 

#### 4.2.1. Synthesis of Acid-Degradable Ketal Crosslinker (Compound **2**) 

Compound **1** was prepared as previously reported [27]. Compound **1** (0.83 g, 5.14 mmol, 1 equiv) in 20 mL of dioxane was mixed with 2.60 g of triethylamine (25.70 mmol, 5 equiv) on ice. Then acryloyl chloride (1.86 g, 20.56 mmol, 4 equiv) in 20 mL of dioxane was added dropwise to the mixture with continuous stirring. After the mixture was stirred for 30 min, the crude product was extracted with ethyl acetate and then purified by silica gel chromatography using a gradient from hexane to 1/1 hexane/ethyl acetate to obtain the yellow product (0.48 g, 1.79 mmol, 35% yield). 

#### 4.2.2. Synthesis of Compound **3**

*N*,*N*′-bis(2-aminoethyl)-1,3-propanediamine (2.00 g, 12.48 mmol, 1 equiv) and triethylamine (3.79 g, 37.44 mmol, 3 equiv) were dissolved in 100 mL of methanol. Ethyl trifluoroacetate (4.43 g, 31.20 mmol, 2.5 equiv), which selectively protects primary amine groups in *N*,*N*′-bis(2-aminoethyl)-1,3-propanediamine, was added to the mixture and stirred for 24 h at room temperature. After the reaction solvent was evaporated, the mixture was purified by silica gel chromatography using an eluent gradient from ethyl acetate to methanol to yield the product as a white solid (3.12 g, 8.86 mmol, 71% yield). 

#### 4.2.3. Synthesis of Acid-Degradable PEG-PAK

PAK was synthesized by reacting compound **3** (0.47 g, 1.33 mmol, 1 equiv) with acid degradable ketal crosslinker (compound **2**, 0.36 g, 1.33 mmol, 1 equiv) in 10 mL of dimethylformamide (DMF) via Michael addition conjugation. Afterwards, the mixtures were stirred for 6 days at 80 °C, and the polymer was precipitated into anhydrous diethyl ether and dried under vacuum. The precipitated polymer was dissolved in 10 mL of 1:9 methanol/6 N NaOH solution and stirred for 6 h to deprotect trifluoroacetate groups. The crude polymer was purified by dialysis (MW cutoff = 6 kDa) against DI water at 4 °C. Then, the polymer was dissolved in deionized (DI) water and amine-reactive 2 kDa PEG-NHS (Laysan Bio, Arab, AL, USA) was added to solution for pegylation. After the pegylation, the polymer was purified by a PD-10 desalting column (MW cutoff = 5 kDa), followed by flash dialysis against DI water (MW cutoff = 6 kDa). The final product was obtained as a powder with a hint of yellow color after lyophilization (0.18 g). Figure 1A shows a summary of the total synthetic process.

### 4.3. Transmission Electron Microscopy (TEM)

One microgram of *SDF-1α* pDNA was complexed with 20 μL of PEG-PAK in DI water (N/P ratio = 100). Two microliters of mixture solution was dropped on a carbon-coated copper TEM grid and air dried for 10 min at room temperature. To observe size and morphological changes of the polyplexes upon acidic condition, 18 μL of *SDF-1α* pDNA/PEG-PAK polyplex solution was mixed with 36 μL of pH 5.0 buffer and further incubated for 8 h at 37 °C. Then 2 μL of polyplex solution was deposited on a TEM grid and dried. TEM images were acquired using a FEI Tecnai G2 Spirit microscope (FEI Company, Hillsboro, OR, USA) operated at 120 kV. 

### 4.4. hADSCs Culture

hADSCs were purchased (Lonza, Basel, Switzerland) and cultured in Dulbecco’s Modified Eagle Medium (Gibco BRL, Gaithersburg, MD, USA) supplemented with 100 units/mL of penicillin (Gibco BRL), 10% (*v*/*v*) fetal bovine serum (FBS, Gibco BRL), and 100 μg/mL of streptomycin (Gibco BRL). All experiments were performed using hADSCs within five passages. To induce hypoxic condition in vitro, hADSCs were cultured under 1% oxygen and serum-free medium, as previously described [28].

### 4.5. In Vitro SDF-1α Transfection

hADSCs were seeded at a density of 3 × 10^5^ cells/well 24 h prior to transfection. *SDF-1α* pDNA PEG-PAK polyplexes were prepared by mixing 8 μg (=600 pmol) of *SDF-1α* pDNA dissolved in 300 μL of DI water with a desired amount of PEG-PAK polymer in 300 μL of DI water to obtain the N/P ratio of 100. *SDF-1α* pDNA/PEI polyplexes at the N/P ratio of 10 were prepared as a control. After 30 min of incubation at room temperature, the culture medium in the dish was replaced with 5.4 mL of FBS-free Dulbecco Modified Eagle Medium‎ (DMEM). Then 600 μL of polyplex solution (or naked SDF-1α pDNA) was added to the cells to obtain a final concentration of 100 nM. After 4 h of incubation, the medium in the dish was replaced with fresh medium containing 10% FBS, followed by further incubation for 48 h.

### 4.6. Confocal Laser Scanning Microscopy

For fluorescence labeling of pDNA/PEG-PAK polyplexes, the desired amount of PEG-PAK (N/P = 100) was mixed with 5 μg of Alexa Fluor 488-labeled DNA in DI water (final volume of 500 μL). After 30 min of incubation, the polyplexes were labeled with amine-reactive Cy3 dye by following the manufacturer’s instructions. Labeled pDNA/ PEG-PAK polyplexes were purified by a PD-10 size exclusion column. hADSCs were seeded at a density of 2 × 10^4^ cells/well in a Falcon 8-well culture slide, 15 h prior to the incubation with the fluorescently labeled polyplexes. After the medium was replaced with 250 μL of fresh DMEM, 50 μL of polyplex solution containing 0.5 μg of Alexa Fluor 488-labeled DNA was incubated with the cells in a well. After 8 h of incubation, the cells were washed with PBS, followed by fixation with 2% paraformaldehyde for 30 min at 4 °C. Then the nuclei of the cells were counter-stained with 1 μM DRAQ5 solution in PBS. The intracellular localization and disassembly of pDNA/PEG-PAK polyplexes in hADSCs were observed under a confocal laser scanning microscope (Olympus Fluoview 500, Olympus America, Melville, NY, USA) using a 60× water immersion Plan-Apochromat objective lens. Fluorescence images of Alexa Fluor 488-labeled pDNA (green) were acquired using a 488 nm excitation light from a multiple argon laser. A krypton laser with 568 nm excitation was used to scan the images of the Cy3-labeled PEG-PAK (red). Images of DRAQ5-stained nuclei (blue) were acquired using a 633 nm helium-neon laser. Images were scanned at mid z-axis point of the cells to differentiate the fluorescence from the polyplexes adsorbed on the cell surface.

### 4.7. Reverse Transcription-Polymerase Chain Reaction (RT-PCR)

hADSCs were homogenized and lysed in TRIzol reagent. Total ribonucleic acid (RNA) was extracted from hADSCs 5 days after hypoxic culture using chloroform. After the extracted RNA was precipitated with 80% (*v*/*v*) isopropanol, the RNA pellet was washed with 75% (*v*/*v*) ethanol, air-dried, and dissolved in 0.1% (*v*/*v*) diethyl pyrocarbonate-treated water. RNA concentration was determined by measuring the absorbance at 260 nm using a spectrophotometer. Reverse transcription was performed using 5 μg of pure total RNA and SuperScriptTM II reverse transcriptase, followed by PCR amplification of the synthesized complementary deoxyribonucleic acid. The deoxyribonucleic acid concentration of all the samples in the different experimental sets was measured with a NanoDrop ND-1000 (NanoDrop Technologies, Rockland, Denmark). PCR consisted of 35 cycles of denaturing (94 °C, 30 s), annealing (58 °C, 45 s), and extension (72 °C, 45 s), with a final extension at 72 °C for 10 min. PCR was followed by electrophoresis on a 2% (*w*/*v*) agarose gel and visualization by ethidium bromide staining. PCR products were analyzed using a gel documentation system (Gel Doc 1000, Bio-Rad, Hercules, CA, USA). *β-actin* served as an internal control.

### 4.8. Western Blot Analysis

The retrieved samples were lysed in ice-cold lysis buffer (15 mM Tris·HCl (pH 8.0), 0.25 M sucrose, 15 mM NaCl, 1.5 mM MgCl_2_, 2.5 mM ethylenediaminetetraacetic acid (EDTA), 1 mM ethylene glycol tetraacetic acid (EGTA), 1 mM DTT, 2 mM NaPPi, 1 μg/mL pepstatin A, 2.5 μg/mL aprotinin, 5 μg/mL leupeptin, 0.5 mM phenylmethylsulfonyl fluoride, 0.125 mM Na_3_VO_4_, 25 mM NaF, and 10 μM lactacystin). After protein concentration was determined using a bicinchoninic acid assay (BCA) kit, equal protein concentration from each sample as mixed with Laemmli sample buffer, loaded, and separated by sodium dodecyl sulfate-polyacrylamide gel electrophoresis (SDS-PAGE) on a 10% (*v*/*v*) resolving gel. Proteins separated by SDS-PAGE were applied to an Immobilon-P membrane (Millipore Corp., Billerica, MA, USA) and then probed with antibody against SDF-1α (Abcam, Cambridge, UK) for 1 hour at room temperature. The membranes were incubated with horseradish peroxidase-conjugated secondary antibody (Santa Cruz Biotechnology, Santa Cruz, CA, USA) for 1 hour at room temperature. The blots were developed using an enhanced chemiluminescence detection system (Amersham Bioscience, Piscataway, NJ, USA). Luminescence was recorded on X-ray film (Fuji super RX, Fujifilm Medical Systems, Tokyo, Japan), and bands were detected using a densitometer (Model GS-690, BioRad, Hercules, CA, USA).

### 4.9. Enzyme-Linked Immunosorbent Assay (ELISA)

Concentrations of angiogenic growth factors in conditioned media obtained from hADSCs cultures were determined using ELISA kits for human SDF-1α, VEGF, and FGF2 (R&D Systems, Minneapolis, MN, USA) according to the manufacturer’s protocol. 

### 4.10. Model of Mouse Hindlimb Ischemia

Hindlimb ischemia was induced in mice as previously described [29]. Four-week old female athymic mice (20–25 g body weight, Bar Harbor, ME, USA) were anesthetized with xylazine (10 mg/kg) and ketamine (100 mg/kg). The femoral artery and its branches were ligated via skin incision using a 6-0 silk suture (Ethicon, Somerville, NJ, USA), along with the external iliac artery and all upstream arteries. The femoral artery was then excised from its proximal origin as a branch of the external iliac artery to the distal point whereupon it bifurcates into the saphenous and popliteal arteries. The use and care of all animals in this study were approved by the Institutional Animal Care and Use Committee of Washington University in St. Louis Medical School (No. 20110260, 6 February 2011) and Sungkyunkwan University (No. SKKUIACUC-17-5-3-3, 3 May 2017).

### 4.11. Transplantation of SDF-1α Transfected hADSC into Ischemic Mouse Hindlimbs

After arterial dissection, mice were randomly divided into six groups (*n* = 8 for each group). Naked *SDF-1α* pDNA, hADSC, *SDF-1* pDNA/PEI transfected hADSCs, or *SDF-1* pDNA/PEG-PAK transfected hADSC (3 × 10^6^ cells/limb) were intramuscularly injected into the gracilis muscle of the medial thigh.

### 4.12. Histology and Laser Doppler Imaging Analysis

Ischemic limb muscles retrieved 21 days post-treatment were fixed with a formaldehyde solution, dehydrated with a graded ethanol series, and embedded in paraffin. Next, 4-µm sections obtained from the specimens were stained with hematoxylin and eosin (H&E) to examine muscle degeneration and tissue inflammation. Laser Doppler imaging analysis (LDPI) was performed as described previously [29]. A laser Doppler perfusion imager (Moor Instruments, Devon, UK) was used for serial noninvasive physiological evaluation of neovascularization. Mice were monitored by serial scanning of surface blood flow in hindlimbs on days 0, 7, 14, and 21 after treatment. Digital color-coded images were scanned and analyzed to quantify a blood flow in ischemic regions from the knee joint to the toe. Mean values of perfusion were subsequently calculated.

### 4.13. Immunohistochemistry

Ischemic limb muscles harvested 21 days post-treatment were embedded in optimal cutting temperature compound (O.C.T. compound, TISSUE-TEK^®^ 4583, Sakura Finetek USA Inc., Torrance, CA, USA), followed by freezing and slicing into 10 µm-thick sections at −22 °C. After all the samples were completely sectioned, ten slides were selected out of the beginning, middle, and end part of each sample. Immunofluorescent staining with anti-human nuclear antigen (HNA) (Chemicon, Temecula, CA, USA) was conducted to detect transplanted human cells from the sections. Immunofluorescent staining with caspase-3 (Abcam, Cambridge, UK) was used to detect apoptotic cells. For the detection of capillaries and arterioles in ischemic regions, sections were immunofluorescently stained with anti-CD31 (PECAM, Abcam) and anti-smooth muscle (SM) α-actin (Abcam), respectively, followed by being examined using a fluorescent microscope (Nikon TE2000, Tokyo, Japan). Twenty different images per slide were randomly acquired from three different samples and analyzed at ×200 magnification. CD31-positive vessels with single-layered round morphology and SM α-actin-positive vessels with multiple-layered round morphology were counted, respectively. Fluorescent vessels with round morphology were counted and calculated as vessel number per mm^2^. Rhodamine- (red) or FITC- (green) conjugated secondary antibodies (Jackson ImmunoResearch Laboratories, West Grove, PA) were used to visualize the stained vessels. Cellular nuclei were counter-stained with DAPI (Vector Laboratories, Burlingame, CA).

### 4.14. Statistical Analysis

GraphPad Prism 7 Software (GraphPad Prism 7, GraphPad Software, San Diego, CA, USA) was used for performing statistical analysis. Triplicate data were analyzed using with one-way analysis of variance (ANOVA) with a Bonferroni test for comparing more than two groups in all experiments (Figure 1, Figure 2, Figure 3, Figure 4, Figure 5 and Figure 6). A *p*-value of <0.05 was considered to be significant. Data are presented as mean with standard deviation for all the measurements.
